# Hedgehog Suppresses Paclitaxel Sensitivity by Regulating Akt-Mediated Phosphorylation of Bax in EGFR Wild-Type Non-Small Cell Lung Cancer Cells

**DOI:** 10.3389/fphar.2022.815308

**Published:** 2022-02-18

**Authors:** Yun-Chieh Tu, Wei-Chen Yeh, Hsin-Hsien Yu, Yu-Cheng Lee, Bor-Chyuan Su

**Affiliations:** ^1^ School of Medicine, College of Medicine, Taipei Medical University, Taipei, Taiwan; ^2^ Division of General Surgery, Department of Surgery, School of Medicine, College of Medicine, Taipei Medical University, Taipei, Taiwan; ^3^ Division of General Surgery, Department of Surgery, Wan Fang Hospital, Taipei Medical University, Taipei, Taiwan; ^4^ Graduate Institute of Medical Sciences, College of Medicine, Taipei Medical University, Taipei, Taiwan; ^5^ Department of Anatomy and Cell Biology, School of Medicine, College of Medicine, Taipei Medical University, Taipei, Taiwan

**Keywords:** Hedgehog, EGFR, paclitaxel, GDC-0449, non-small cell lung cancer

## Abstract

Non-small cell lung cancer (NSCLC) is one of the most common and deadly cancers worldwide. Among NSCLC patients, almost half have wild-type epidermal growth factor receptor (EGFR WT). The primary therapeutic option for these EGFR WT NSCLC patients is chemotherapy, while NSCLC patients with EGFR mutations have more diverse therapeutic options, including EGFR tyrosine kinase inhibitors. Moreover, NSCLC patients with EGFR WT have worse chemotherapy response than EGFR mutant NSCLC patients. Thus, an urgent need exists for novel therapeutic strategies to improve chemotherapy response in EGFR WT NSCLC patients. Hedgehog signaling is known to be highly active in NSCLC; however, its potential role in chemoresistance is not fully understood. In the present study, we found that paclitaxel (PTX) treatment induces hedgehog signaling in EGFR WT NSCLC cells, and inhibition of hedgehog signaling with GDC-0449 (Vismodegib) increases sensitivity to PTX-stimulated apoptosis. Furthermore, GDC-0449 potentiates PTX-induced reactive oxygen species and mitochondrial dysfunction. In contrast, a hedgehog agonist, Hh-Ag1.5, attenuates PTX-induced apoptosis. Mechanistic experiments revealed that hedgehog induces phosphorylation of Akt at Ser473. Akt then phosphorylates Bax at Ser184, which can switch its activity from pro-apoptosis to anti-apoptosis. Taken together, our findings suggest that inhibition of hedgehog signaling might be a promising therapeutic strategy to improve PTX response in EGFR WT NSCLC.

## Introduction

Non-small cell lung cancer (NSCLC) is the most common type of lung cancer worldwide (Yuan et al., 2019), and its main treatment options are surgical resection and chemotherapy (Felip et al., 2010). Currently, the chemotherapeutic agents used for NSCLC include platinum-based drugs, gemcitabine, docetaxel, and paclitaxel (PTX) (Zappa and Mousa, 2016; Pirker, 2020). In addition, epidermal growth factor receptor (EGFR)-targeted therapy and immunotherapy may exhibit therapeutic benefits for some patients (Zappa and Mousa, 2016; Pirker, 2020). However, EGFR tyrosine kinase inhibitors (TKIs) only provide clinical benefits in patients who carry specific EGFR mutations (Tomasini et al., 2017; Pirker, 2020). Almost half of NSCLC patients show no EGFR mutation (EGFR WT) (Xu et al., 2015), leaving chemotherapy and immunotherapy as the only viable therapeutic options (Tomasini et al., 2017). Clinical trials have demonstrated anti-PD-1/anti-PD-L1 therapies can prolong overall survival in EGFR WT NSCLC patients (To et al., 2021), and chemotherapy combined with immunotherapy was approved by the United States FDA in 2020 for the treatment of metastatic or recurrent NSCLC (Shields et al., 2021). Nevertheless, the utility of these therapies is limited by adverse effects of anti-PD-1/anti-PD-L1 treatments (Su et al., 2020) and the high prices for immunotherapeutic drugs (Green, 2021). Therefore, despite the recent advances in developing novel treatments for NSCLC, chemotherapy remains the main therapeutic option for most patients (Vasekar et al., 2020; Green, 2021). As patients receiving chemotherapy often develop resistance to the drugs, chemoresistance represents the main cause of therapeutic failure in NSCLC (Iglesias et al., 2018). To make matters worse, EGFR WT NSCLC patients also have poor initial response to chemotherapy (Liang et al., 2014). For these reasons, the 5-years survival rate for the disease is still below 21% (Lu et al., 2019), and new cost-effective therapeutic strategies are urgently needed to overcome poor chemotherapy response in EGFR WT NSCLC patients.

Hedgehog signaling is crucial for embryonic development and adult tissue homeostasis (Giroux-Leprieur et al., 2018). Activation of the canonical hedgehog pathway depends on hedgehog-ligand receptor interactions (Giroux-Leprieur et al., 2018). When sonic hedgehog (Shh) blinds to the patched receptor, glioma-associated oncogene homolog 1 (GLI1) dissociates from suppressor of fused protein (Giroux-Leprieur et al., 2018). The free GLI1 subsequently enters the nucleus where it regulates its target genes (Giroux-Leprieur et al., 2018). On the other hand, activation of the non-canonical hedgehog pathway can be directly triggered by EGFR, transforming growth factor-β, and tumor necrosis factor-α pathway (Giroux-Leprieur et al., 2018). Under normal conditions, hedgehog is silenced in lung tissues. However, GLI1 and Shh are overexpressed in human NSCLC, and their expression levels are significantly correlated with tumor recurrence (Giroux-Leprieur et al., 2018). Furthermore, hedgehog has been implicated in lung cancer chemoresistance and radioresistance (Giroux-Leprieur et al., 2018). A case report demonstrated that vismodegib (GDC-0449; GDC), a hedgehog pathway inhibitor approved for the treatment of metastatic basal cell carcinoma, also exhibits promising anticancer potential in NSCLC patients (Tsao et al., 2017). This case report suggested that hedgehog signaling could be a potential therapeutic target in NSCLC. However, the detailed mechanisms by which hedgehog signaling affects chemotherapy sensitivity in lung cancer are not fully known. Nevertheless, it is known that lung cancer patients with high levels of phosphorylated Akt have poor prognosis and do not respond well to TKIs (Tang et al., 2006; Jacobsen et al., 2017). Moreover, the hedgehog pathway was recently linked to activation of phosphoinositide 3-kinase (PI3K)/Akt in leukemia cells (Kern et al., 2015). Despite this potential mechanistic connection, it remains unclear whether PI3K/Akt signaling is directly modulated by hedgehog in lung cancer cells. In this study, we delineated the role of hedgehog in PTX sensitivity in EGFR WT NSCLC cells. Our results suggest a novel therapeutic approach to alleviate resistance to PTX in EGFR WT NSCLC.

## Materials and Methods

### Reagents

4′,6-diamidino-2-phenylindole dihydrochloride (DAPI), 2′,7′-dichlordihydrofluorescein diacetate (DCFDA), dihydroethidium (DHE), mitoTEMPO (TEMPO), phenazine methosulfate (PMS), and paclitaxel (PTX) were purchased from Merck (Darmstadt, Germany). Tetramethylrhodamine, ethyl ester (TMRE) was purchased from Thermo Fisher Scientific (Waltham, United States). GDC-0449 (GDC) and Hh-Ag1.5 were purchased from BioVision (Milpitas, United States). MTS was purchased from Promega (Madison, United States). Z-VAD-FMK was purchased from Cell Signaling Technology (Danvers, United States). FITC Annexin V was purchased from BioLegend (San Diego, United States).

### Cell Culture

The A549 NSCLC cell line and MRC-5 non-cancerous human lung cell line were purchased from BCRC (Hsinchu, Taiwan). A549 and MRC-5 cells were maintained in DMEM (Thermo Fisher Scientific; Waltham, United States) supplemented with 10% fetal bovine serum (Peak; Colorado, United States) and antibiotic-antimycotic (Thermo Fisher Scientific; Waltham, United States). The H1299 cell line (an EGFR WT NSCLC cell line) was kindly provided by Dr. Pan-Chyr Yang (Institute of Biomedical Sciences, Academia Sinica). The H1299 line was maintained in RPMI (Thermo Fisher Scientific; Waltham, United States) supplemented with 10% fetal bovine serum (Peak; Colorado, United States) and antibiotic-antimycotic (Thermo Fisher Scientific; Waltham, United States).

### Cell Viability and Apoptotic Assay

The trypan blue exclusion assay was performed to assess cell viability. Briefly, both suspended cells and adherent cells were collected and mixed with trypan blue. Viable and dead cells were observed under light microscopy. The MTS/PMS activity assay was also performed according to the manufacturer’s instructions to assess cell viability. Apoptosis was assessed as previously described, according to chromatin condensation (Su and Mo, 2014) and annexin V staining (Su et al., 2019). In addition, the TUNEL assay was performed according to the manufacturer’s protocol (AAT Bioquest). After TUNEL staining, apoptotic cells in five random fields of view were scored under fluorescence microscopy.

### Immunofluorescence Assay

After treatments, cells were fixed in methanol at −20°C for 10 min and permeabilized with cold methanol. Thereafter, cells were blocked with 0.1% bovine serum albumin in PBS at room temperature for 1 h and then stained with Alexa Fluor 488 conjugated α-tubulin antibody (Cell Signaling Technology) at 4°C overnight. Cells were then washed with PBS three times and stained with DAPI (Merck). Mitotic morphology was observed under fluorescence microscopy.

### Western Blotting and Antibodies

Antibodies against GLI1, Shh, phospho-Akt (Ser473), Akt, phospho-ERK (Thr202/Tyr204), ERK, cIAP1, XIAP, survivin, caspase-3, PARP, Bax, Bak, catalase, UCP2, SOD1, β-actin were purchased from Cell Signaling Technology (Danvers, United States). Phospho-Bax (Ser184) antibody was purchased from ThermoFisher Scientific (Waltham, United States). Whole cell lysates were collected in RIPA buffer. Protein lysates were separated by gradient SDS-PAGE, and transferred onto PVDF membranes. Thereafter, PVDF membranes were probed with indicated antibodies by standard methods**.**

### Mitochondrial Membrane Potential and ROS Analysis

Mitochondrial membrane potential was validated using TMRE (100 nM). ROS levels was assessed using fluorescent ROS indicators, DHE (20 μM) and DCFDA (10 μM). Briefly, cells were stimulated with indicated treatments, followed by addition of TMRE or fluorescent ROS indicators (DCFDA and DHE) for 15 min. Thereafter, cells were rinsed with phosphate buffered saline three times. TMRE, DCFDA, and DHE intensities were assessed by flow cytometry (Beckman Coulter) or observed under fluorescence microscopy (EVOS FL Cell Imaging System, ThermoFisher, Waltham, United States).

### Statistical Analysis

All experiments were performed as least three times. Results are shown as mean ± SEM. Statistical differences were assessed by Student’s t-test or one way ANOVA using Graph Pad Prism8. *p* values smaller than 0.05 were considered statistically significant.

## Results

### Hedgehog Pathway Is Activated by PTX in EGFR WT NSCLC A549 Cells

**FIGURE 1:**
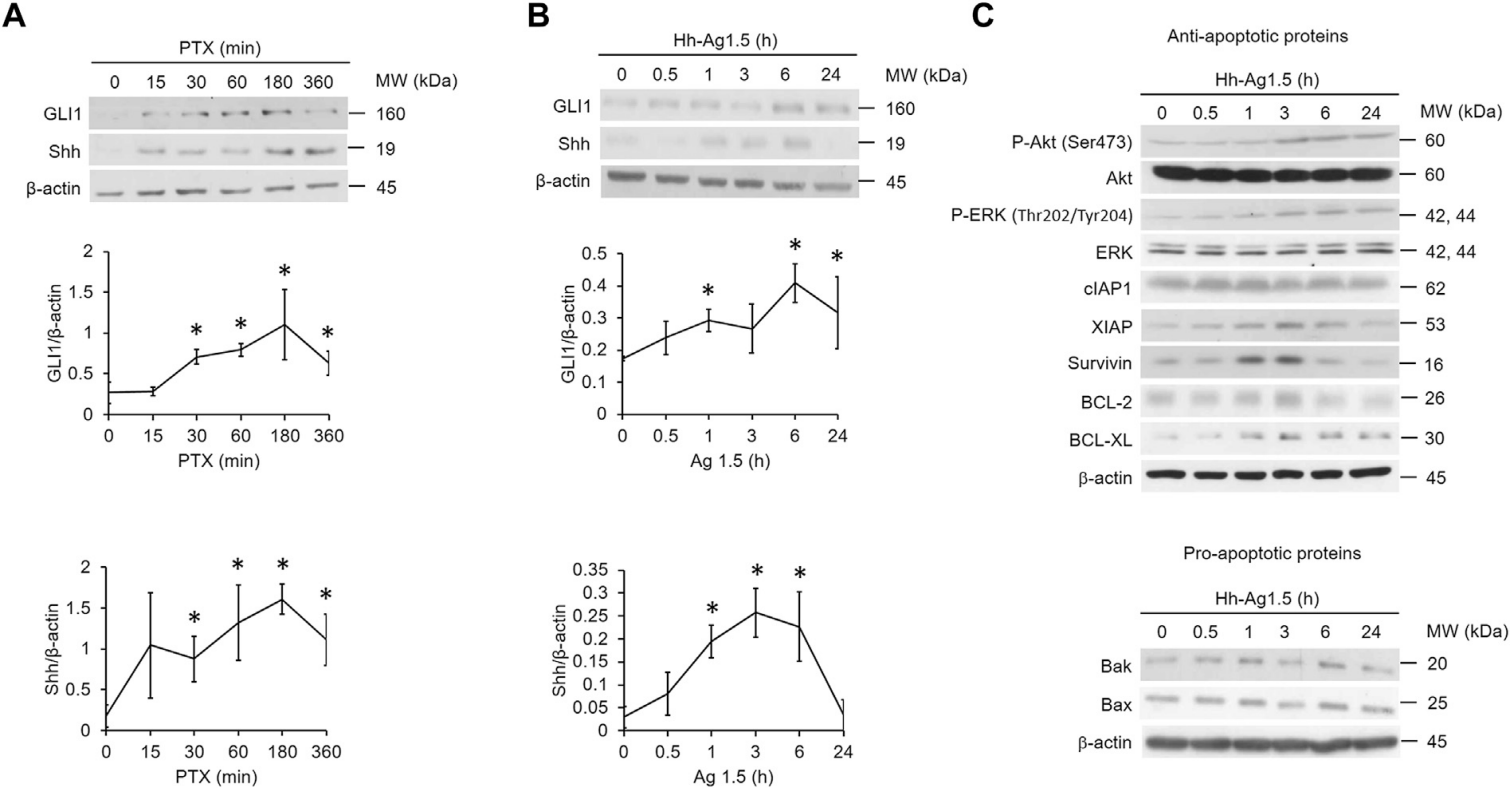
Hedgehog signaling is induced by paclitaxel. **(A,B,C)** A549 cells were treated with paclitaxel (PTX; 20 nM) or Hh-Ag1.5 (1 nM) for the indicated times. Then, total cell lysates were immunoblotted using anti-GLI1, anti-Shh, anti-P-Akt, anti-Akt, anti-P-ERK, anti-ERK, anti-XIAP, anti-survivin, anti-cIAP1, anti-BCL-2, anti-BCL-XL, anti-Bax, anti-Bak, and anti-β-actin antibodies. Protein band intensities were analyzed using ImageJ. *: *p* < .05; indicates statistically significant difference between 0 h or 0 min and the indicated time point. All experiments were performed at least three times. *n* = 3 for all groups.

To test whether the hedgehog pathway is activated upon PTX treatment in EGFR WT NSCLC cells, A549 cells were treated with PTX for various times. Two markers of hedgehog signaling activation, GLI1 and Shh, were monitored (Ma et al., 2013). Both GLI1 and Shh were elevated within 30 min (Figure 1A) of treatment, indicating that the hedgehog pathway is indeed activated by PTX. To determine what actions PTX-induced hedgehog signaling might have in EGFR WT NSCLC cells, the cells were incubated with hedgehog agonist Hh-Ag1.5. As expected, we found that Hh-Ag1.5 induced activation of GLI1 and Shh within 1 h (Figure 1B). We also found that it activated several anti-apoptotic proteins (Figure 1C), as the levels of XIAP, survivin, BCL-2 and BCL-XL peaked at about 3 h after treatment. In addition, Hh-Ag1.5 induced sustained phosphorylation of Akt and ERK, while it had no observable effect on the levels of pro-apoptotic proteins, such as Bax and Bak (Figure 1C).

### Pharmacological Inhibition of Hedgehog Signaling Potentiates PTX-Induced Cytotoxicity in EGFR WT NSCLC Cells

**FIGURE 2:**
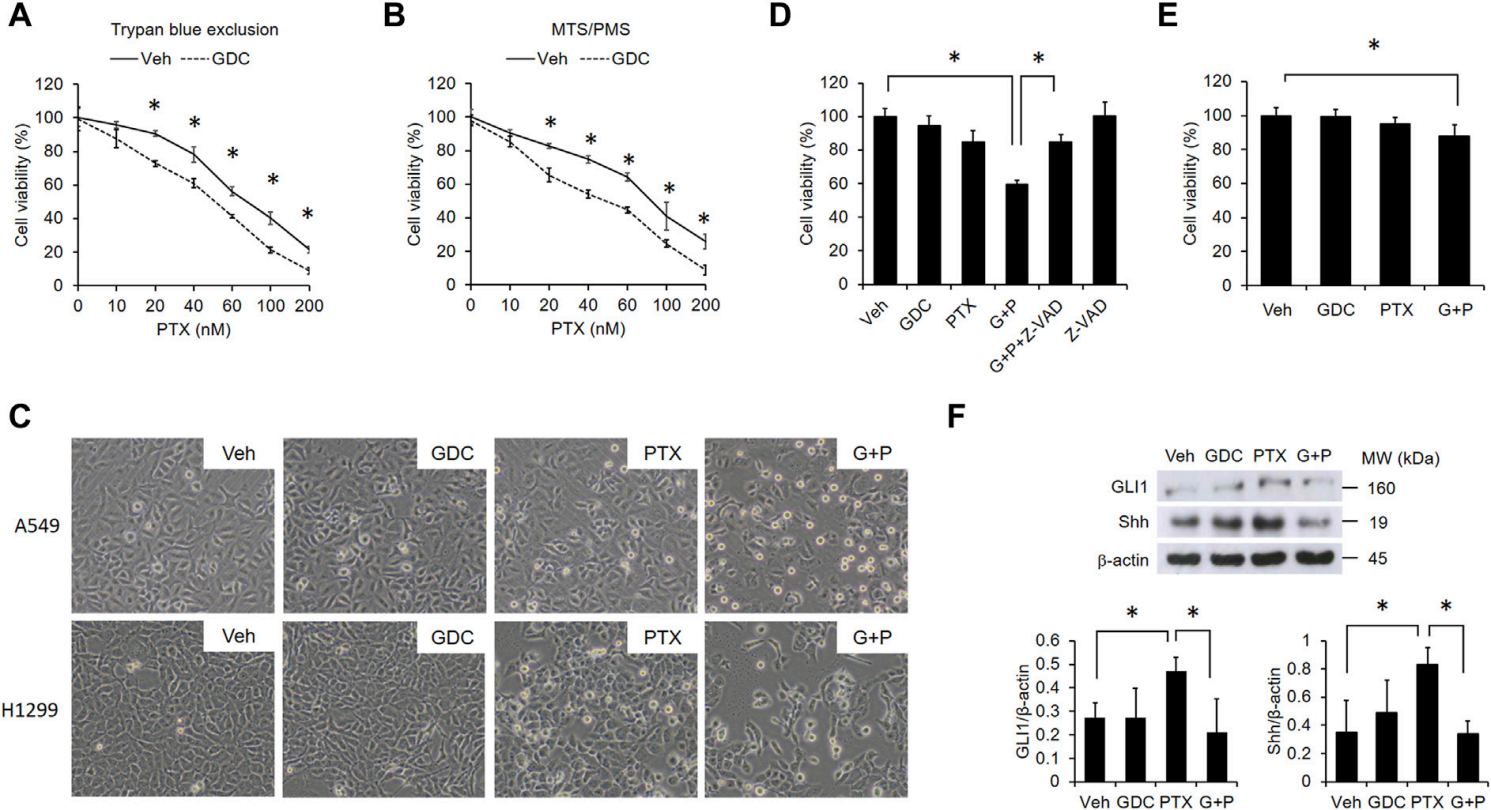
GDC potentiates paclitaxel-induced cytotoxicity in NSCLC cells. A549 cells were preincubated with DMSO (vehicle; Veh) or GDC-0449 (GDC; 20 μM) for 1 h followed by treatment with various doses of PTX for 24 h. Cell viability was determined by trypan blue exclusion **(A)** and MTS/PMS activity **(B)** assays. **(C)** Cells were preincubated with Veh or GDC (20 μM) for 1 h followed by PTX (20 nM) treatment for 24 h. Apoptotic cells were identified by morphology under light microscopy (×20 objective). G + P, GDC + PTX. **(D)** Cells were treated with GDC (20 μM) and PTX (20 nM) for 24 h, with or without preincubation with the pan-caspase inhibitor, Z-VAD-FMK (Z-VAD; 100 μM). G + P, GDC + PTX; G + P + Z-VAD, GDC + PTX + Z-VAD. Cell viability was analyzed by the MTS/PMS activity assay. **(E)** MRC-5 cells were preincubated with Veh or GDC (20 μM) for 1 h followed by PTX (20 nM) treatment for 24 h. Cell viability was assessed with the MTS/PMS assay. **(F)** A549 cells were preincubated with GDC (20 μM) for 1 h followed by PTX (20 nM) treatment for an additional 1 h. Whole cell lysates were collected and immunoblotted with anti-GLI1, anti-Shh, and anti-β-actin antibodies. Protein band intensities were analyzed using ImageJ. G + P, GDC + PTX. *: *p* < .05; indicates a statistically significant difference between indicated groups. All experiments were performed at least three times. *n* = 3 for all groups.

Because PTX induced hedgehog signaling that activates several anti-apoptotic proteins in A549 cells (Figure 1C), we suspected that activation of hedgehog signaling may attenuate PTX-induced cytotoxicity in EGFR WT NSCLC cells. Thus, we preincubated A549 cells with the hedgehog inhibitor, GDC-0449 (GDC). Then, we treated the cells with different doses of PTX. We found that GDC could potentiate PTX-induced cytotoxicity (Figures 2A,B). Furthermore, GDC enhanced PTX-induced apoptosis in EGFR WT NSCLC A549 and H1299 cells, as assessed by rounded cell morphologies (Figure 2C). Cytotoxicity was only significantly induced in the PTX/GDC group compared to the vehicle group (Veh vs PTX, *p* = 0.1686; Veh vs GDC, *p* = 0.7969; Veh vs PTX/GDC, *p* = 0.005). Treatment with the pan-caspase inhibitor, Z-VAD-FMK, abolished the cytotoxicity of GDC combined with PTX in A549 cells (PTX/GDC/Z-VAD vs Veh, *p* = 0.0965), indicating that the combination mainly induces apoptotic cell death (Figure 2D). Notably, there was no statistically significant difference between vehicle and PTX group, and the combination of PTX and GDC showed only marginal cytotoxicity in MRC-5 non-cancerous lung cells (Figure 2E). Moreover, the PTX-induced activation of hedgehog signaling could be inhibited by GDC treatment (Figure 2F).

### GDC Augments PTX-Induced Apoptosis

**FIGURE 3:**
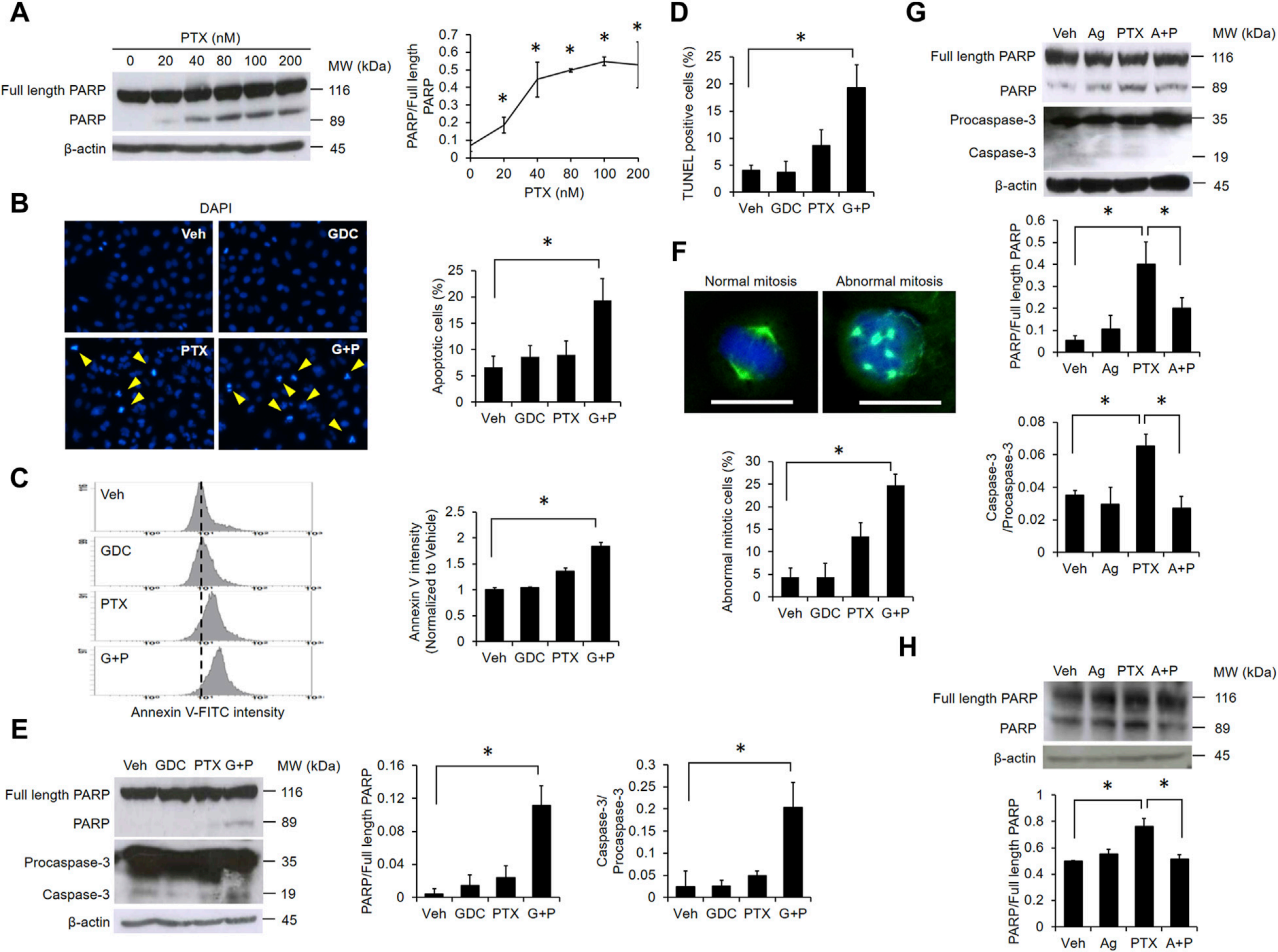
GDC potentiates paclitaxel-induced apoptosis. **(A)** A549 cells were treated with indicated doses of PTX for 24 h. Whole cell lysates were immunoblotted with anti-PARP and anti-β-actin antibodies. Protein band intensities were analyzed using ImageJ. A549 cells were preincubated with GDC (20 μM) for 1 h followed by PTX (20 nM) treatment for 24 h. Chromosome condensation was observed under fluorescence microscopy **(B)**. Phosphatidylserine exposure was assessed using annexin V-FITC staining by flow cytometry **(C)**. DNA double strand breaks were observed using TUNEL assay analyzed with fluorescence microscopy **(D)**. Activation of PARP and caspase-3 were monitored by western blotting **(E)**. A549 cells were treated as in **(B)**; cell mitosis was observed under fluorescence microscopy. Green color: microtubule; blue color: cell nuclei. Scale bar: 20 μm **(F)**. **(G)** A549 cells were preincubated with Hh-Ag1.5 (1 nM) for 24 h followed by PTX (20 nM) treatment for 24 h. Total cell lysates were immunoblotted with anti-PARP, anti-caspase-3, and anti-β-actin antibodies. Protein band intensities were analyzed using ImageJ. **(H)** H1299 cells were preincubated with Hh-Ag1.5 (1 nM) for 24 h followed by PTX (20 nM) treatment for 24 h. Total cell lysates were immunoblotted with anti-PARP and anti-β-actin antibodies. Protein band intensities were analyzed using ImageJ. *: *p* < .05; indicates a statistically significant difference between indicated groups. All experiments were performed at least three times. *n* = 3 for all groups.

PTX dose-dependently induced poly (ADP-ribose) polymerase (PARP) cleavage, further suggesting that PTX induces apoptosis in EGFR WT NSCLC cells (Figure 3A). Therefore, we next validated the apoptotic effect of the PTX/GDC combination by monitoring various markers of apoptosis, including chromosome condensation (Figure 3B), phosphatidylserine exposure (Figure 3C), DNA strand breaks (Figure 3D), and cleavage of PARP and caspase-3 (Figure 3E). Each apoptotic marker was clearly induced in cells treated with the PTX/GDC combination (Figures 3B–E). Furthermore, the percentage of cells with abnormal mitotic spindles was dramatically increased by treatment with the PTX/GDC combination (Figure 3F). While normal mitotic cells exhibit bipolar spindles, abnormal mitotic cells often exhibit multipolar spindles (Figure 3F). Importantly, we found that Hh-Ag1.5 alleviated PTX-induced cleavage of PARP and caspase-3 in A549 cells (Figure 3G), and PTX-induced PARP cleavage could also be suppressed by Hh-Ag1.5 in H1299 cells (Figure 3H).

### GDC Alleviates Akt-Mediated Bax Phosphorylation

**FIGURE 4:**
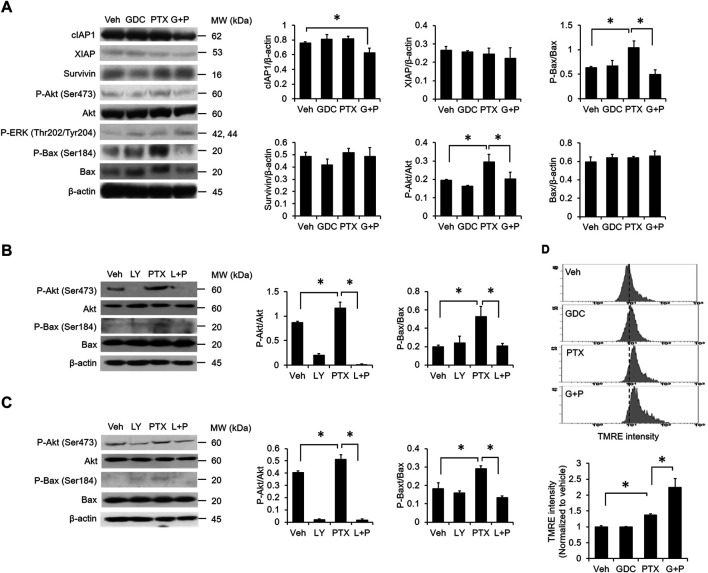
Combination of PTX and GDC reduced Bax phosphorylation. **(A)** A549 cells were preincubated with GDC (20 μM) for 1 h followed by PTX (20 nM) treatment for 24 h. Total cell lysates were immunoblotted using anti-cIAP1, anti-XIAP, anti-survivin, anti-P-Akt, anti-Akt, anti-P-ERK, anti-ERK, anti-P-Bax, anti-Bax, and anti-β-actin antibodies. Protein band intensities were analyzed using ImageJ. *: *p* < .05; indicates a statistically significant difference between indicated groups. A549 **(B)** and H1299 **(C)** cells were preincubated with LY294002 (20 μM) for 1 h followed by PTX (20 nM) treatment for 24 h. Total cell lysates were immunoblotted with anti-P-Akt, anti-Akt, anti-P-Bax, anti-Bax, and anti-β-actin antibodies. Protein band intensities were analyzed using ImageJ. *: *p* < .05; indicates a statistically significant difference between indicated groups. **(D)** A549 cells were preincubated with GDC (20 μM) for 1 h followed by PTX (20 nM) treatment for 24 h. Mitochondrial membrane potential was monitored by TMRE using flow cytometry. All experiments were performed at least three times. *n* = 3 for all groups.

To determine the effects of combined PTX and GDC on anti-apoptotic proteins, A549 cells were preincubated with GDC, followed by treatment with PTX. Western blot results showed that the levels of cIAP were reduced by PTX/GDC (Figure 4A), and GDC also alleviated the PTX-induced phosphorylation of both Akt (Ser473) and Bax (Ser184) (Figure 4A). Of note, the PTX-induced phosphorylation of ERK (Thr202/Tyr204) was not further enhanced or suppressed by GDC (Figure 4A), even though phosphorylation of ERK is slightly induced by GDC alone. A previous study showed that phosphorylated Akt can phosphorylate Bax and switch its function from pro-apoptosis into anti-apoptosis (Kale et al., 2018). We therefore treated cells with the PI3K/Akt inhibitor, LY294002, and found that it could abolish PTX-induced phosphorylation of Akt and Bax in both A549 (Figure 4B) and H1299 (Figure 4C) cells. In addition, GDC potentiated PTX-induced mitochondrial hyperpolarization (Figure 4D).

### Mitochondrial ROS Is Crucial for PTX/GDC-Induced Apoptosis

**FIGURE 5:**
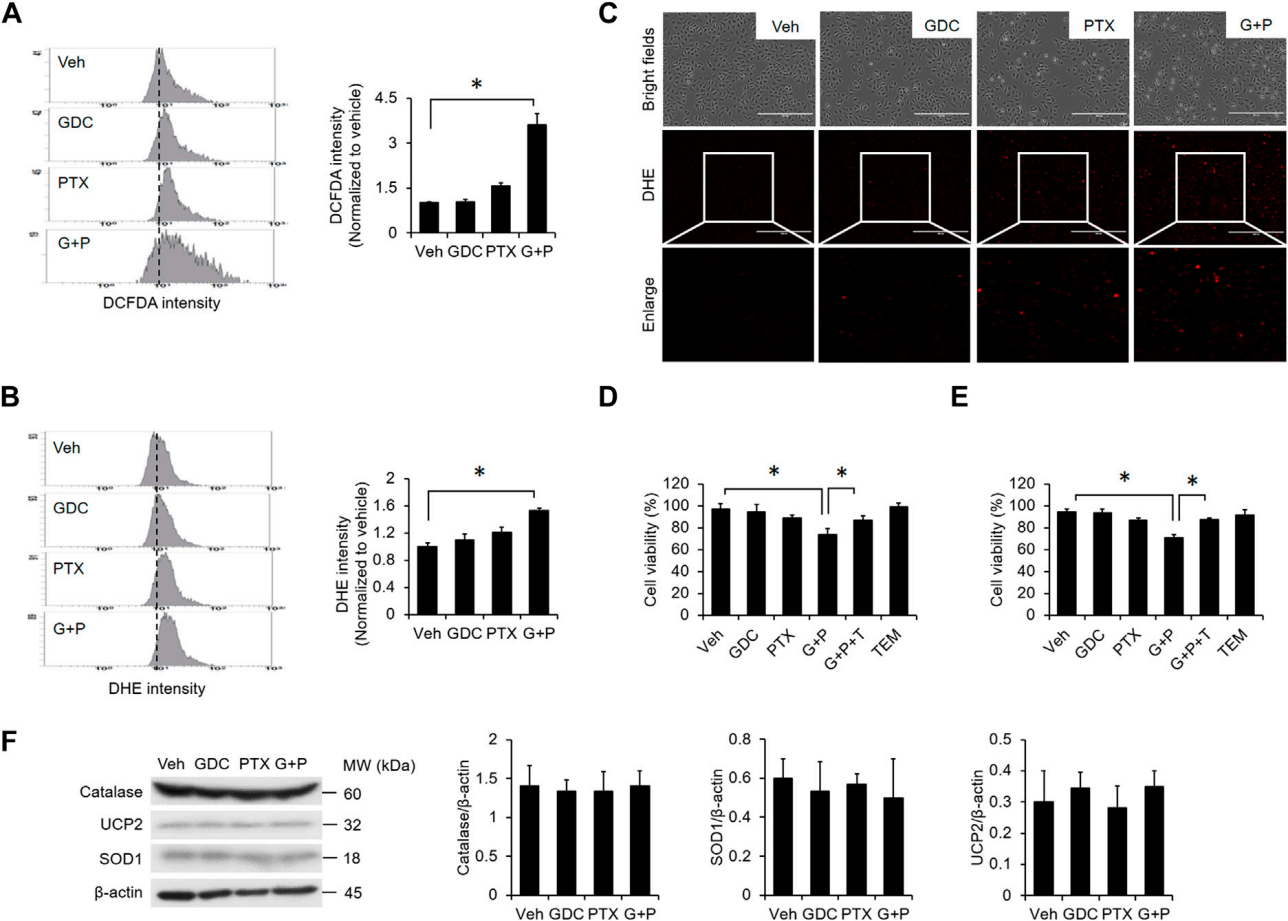
ROS generation is enhanced by the combination of PTX and GDC. A549 cells were preincubated with GDC (20 μM) for 1 h followed by PTX (20 nM) treatment for 24 h. Intracellular ROS levels were monitored by DCFDA **(A)** and DHE **(B,C)**. A549 **(D)** and H1299. **(E)** A549 cells were treated with GDC (20 μM) and PTX (20 nM) for 24 h, with or without preincubation with the mitochondrial ROS scavenger, mitoTEMPO (TEM; 10 μM). Cell viability was assessed by trypan blue exclusion assay. G + P, GDC + PTX; G + P + T, GDC + PTX + TEMPO. **(F)** Cells were treated as in **(A)**; total cell lysates were immunoblotted with anti-catalase, anti-UCP2, anti-SOD1, and anti-β-actin antibodies. G + P, GDC + PTX. Protein band intensities were analyzed using ImageJ. G + P, GDC + PTX. *: *p* < 0.05; indicates a statistically significant difference between indicated groups. All experiments were performed at least three times. *n* = 3 for all groups.

Previous studies demonstrated that the elevation of reactive oxygen species (ROS) plays a crucial role in PTX-induced apoptosis (Meshkini and Yazdanparast, 2012; Huang et al., 2017). We found that PTX treatment alone slightly elevated the intracellular level of ROS (Figures 5A–C), and addition of GDC further enhanced PTX-induced ROS (Figures 5A–C). Moreover, the specific mitochondrial ROS scavenger mitoTEMPO abolished PTX/GDC-induced cell death in both A549 (Figure 5D) and H1299 (Figure 5E) cells, suggesting an essential role for mitochondrial ROS in this process. However, the combination of GDC and PTX did not affect the levels of antioxidant proteins, such as catalase, UCP-2 and SOD1 (Figure 5F).

## Discussion

**FIGURE 6:**
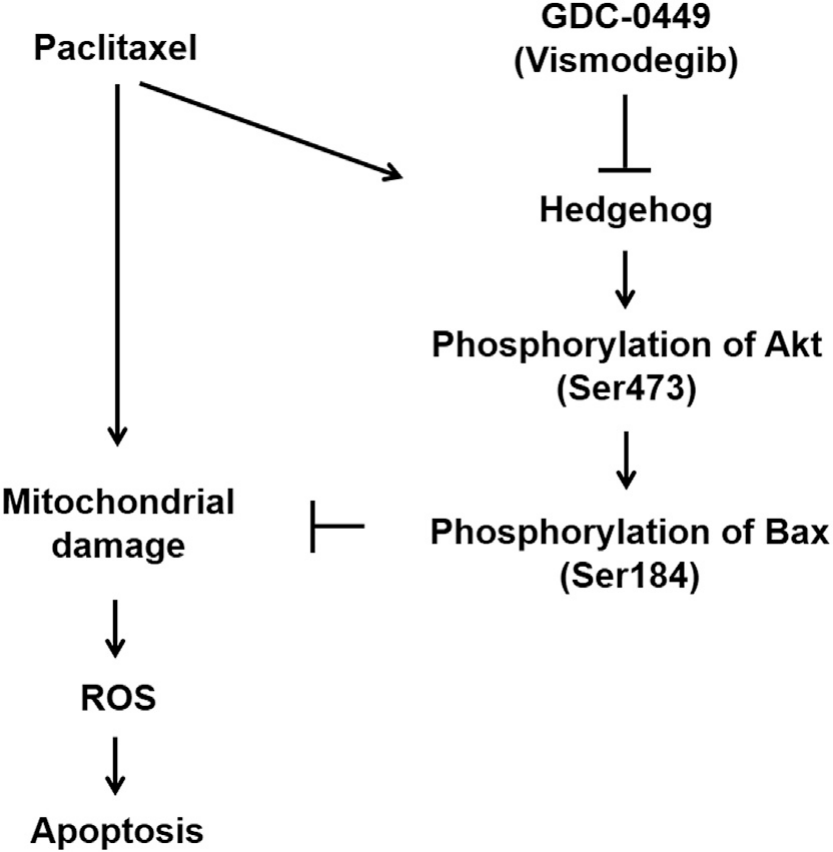
Model of GDC potentiation of PTX-induced apoptosis. Paclitaxel treatment induces apoptosis but is accompanied by activation of the protective hedgehog pathway. Hedgehog induces phosphorylation of Akt (Ser473), which in turn phosphorylates Bax at Ser184. Phosphorylated Bax is unable to translocate to mitochondria. Thus, hedgehog-mediated Bax phosphorylation renders EGFR WT NSCLC cells resistant to paclitaxel-induced apoptosis. GDC-0449 attenuates hedgehog signaling activation, potentiating paclitaxel-induced apoptosis.

In this study, we found that hedgehog signaling is induced by PTX treatment of EGFR WT NSCLC cells (Figure 1). Furthermore, pharmacological inhibition of hedgehog signaling with GDC potentiates PTX-induced mitochondrial damage (Figure 4), ROS accumulation (Figure 5), abnormal mitosis and apoptosis in EGFR WT NSCLC cells (Figure 3), suggesting that hedgehog signaling may play a significant role in modulating PTX sensitivity. Our experiments probing the underlying mechanism revealed that hedgehog induces Akt-mediated inactivation of Bax (Figure 4). Based on these findings, we conclude that a PTX-induced hedgehog-Akt-Bax signaling axis promotes chemoresistance in EGFR WT NSCLC cells (Figure 6).

PTX induces cytotoxicity in various cancer cell types by stabilizing microtubules and subsequently interfering with mitosis (Whitaker and Placzek, 2019; Fang et al., 2021). This mode of action is consistent with our findings (Figure 3F). We found that GDC can enhance PTX-induced mitotic abnormalities, suggesting that hedgehog signaling might prevent the effect. In addition to its effects on microtubules, PTX also targets mitochondria (Andre et al., 2000; Whitaker and Placzek, 2019). BCL-2 is a key inhibitor of the mitochondrial apoptosis pathway, and its high expression levels are associated with poor therapeutic efficacy of PTX in lung cancer patients (Maraz et al., 2011), and attenuated death in multiple myeloma cells (Gazitt et al., 1998). Thus, mitochondria are likely to be central players in the anticancer activity of PTX. In the present study, we found that PTX induces apoptosis in EGFR WT NSCLC cells, but the cell death is accompanied by a competing protective activation of the hedgehog pathway (Figures 1–3). While the induction mechanism in NSCLC cells remains unclear, hedgehog activation can render the cells more resistant to PTX-induced mitochondrial damage and apoptosis by a mechanism involving phosphorylation of Bax at S184 (Figure 4). The apoptotic regulatory activity of Bax is known to be determined by phosphorylation at different residues (Kale et al., 2018). For example, Bax phosphorylation at S184 prevents its insertion into mitochondria, which renders cancer cells resistant to apoptotic stimuli (Kale et al., 2018). In contrast, phosphorylation at Thr167 triggers Bax translocation to mitochondria (Kim et al., 2006). Notably, Akt is essential for phosphorylation of Bax at S184 (Gardai et al., 2004). In line with this mechanism, the activation of hedgehog and Akt in lung cancer are associated with resistance to chemotherapy (Giroux-Leprieur et al., 2018), poor prognosis (Tang et al., 2006), and low survival rate (Tang et al., 2006). We found that Akt phosphorylation is induced by hedgehog agonist Hh-Ag1.5 (Figure 1), suggesting that Akt is a downstream target of hedgehog. Similar findings were reported for glioblastoma (Chang et al., 2015), and it is possible that hedgehog might induce Akt phosphorylation via suppression of PTEN (Pietrobono and Stecca, 2018). Regardless of the precise signaling mechanism, PTX-induced Akt and Bax phosphorylation was effectively inhibited by the addition of GDC (Figure 4). Furthermore, the combination treatment induced robust mitochondrial damage in A549 cells (Figure 4). Together, these findings suggested that hedgehog induces protective Akt-mediated signaling to counteract PTX-induced mitochondrial damage in EGFR WT NSCLC cells.

GDC (Vismodegib) is an FDA-approved drug for the treatment of metastatic basal cell carcinoma, and it acts to inhibit the hedgehog pathway by antagonizing smoothened protein (Babacan et al., 2012). Its therapeutic benefits for several different types of cancers have recently received increased attention. For example, recent studies have demonstrated that GDC treatment exhibits promising anticancer activities in NSCLC (Tsao et al., 2017) and medulloblastoma (Li et al., 2019). In addition, clinical and preclinical studies have explored the utility of combining GDC with standard chemotherapeutic agents or other therapeutic strategies in various types of cancer. As such, GDC in combination with photodynamic therapy exhibits strong antitumor activity in multiple basal cell carcinomas (Rizzo et al., 2018). Additionally, the combination of GDC with standard chemotherapeutic agents exhibits better anti-osteosarcoma action than chemotherapeutic agents alone (Saitoh et al., 2016). However, the addition of GDC to standard chemotherapeutic agents does not always improve outcome. The combinations failed to induce better therapeutic efficacy in metastatic pancreatic adenocarcinoma (De Jesus-Acosta et al., 2020) and extensive-stage small cell lung cancer (Belani et al., 2016). Despite the inconsistent therapeutic benefits of GDC combinations, clinical trials in patients have demonstrated that the combination of GDC with chemotherapeutics is safe (Rizzo et al., 2018). Our study in cultured NSCLC cells suggests that the combination of PTX and GDC has potential to produce better therapeutic response than PTX alone in EGFR WT NSCLC. Whether GDC could also increase the abilities of other chemotherapeutic agents to induce cytotoxicity in NSCLC remains to be explored. It is worthwhile to evaluate the potential for repurposing GDC for NSCLC because GDC is already approved for the treatment of metastatic basal cell carcinoma. Therefore, its clinical safety and pharmacology are already well characterized. Also, the combination of PTX and GDC to treat EGFR WT NSCLC is relatively easy to implement, allowing this strategy to be applied in the near future if successful.

Overall, our study shows that pharmacological inhibition of hedgehog signaling is a promising approach to increase the sensitivity of EGFR WT NSCLC cells to PTX treatment. Our findings further suggest that lower doses of PTX may exhibit anticancer activity when hedgehog signaling is suppressed, potentially through synergistic or additive drug effects. In future studies, we plan to investigate the therapeutic efficacy of this combination *in vivo* using an EGFR WT NSCLC patient-derived xenograft (PDX) model. The PDX model will allow us to extend our *in vitro* findings and test the implications of our data in a whole animal system.

Importantly, our current findings reveal an underlying mechanism of hedgehog signaling-mediated PTX resistance in EGFR WT NSCLC cells, which involves Akt-mediated phosphorylation of Bax. Thus, our study suggests the combination of PTX and GDC might be an effective therapeutic option for EGFR WT NSCLC patients.

## Data Availability

The raw data supporting the conclusion of this article will be made available by the authors, without undue reservation.
