# Supplementary material for: Hedgehog Suppresses Paclitaxel Sensitivity by Regulating Akt-Mediated Phosphorylation of Bax in EGFR Wild-Type Non-Small Cell Lung Cancer Cells
Source: Front Pharmacol. 2022 Feb 18;13:815308. doi: 10.3389/fphar.2022.815308 (PMC8894848; doi:10.3389/fphar.2022.815308)

Figure 1A

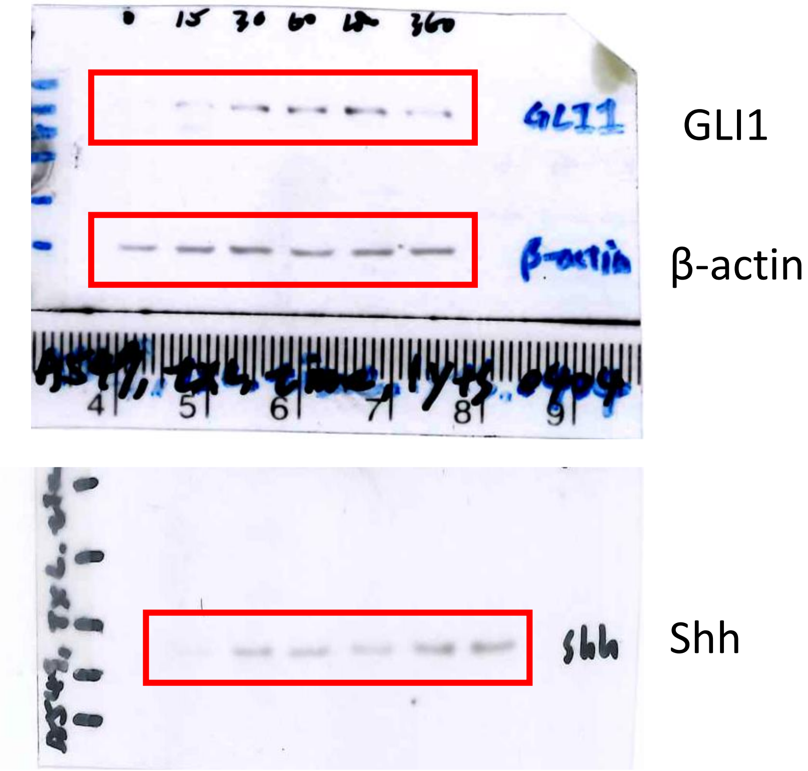

Figure 1B

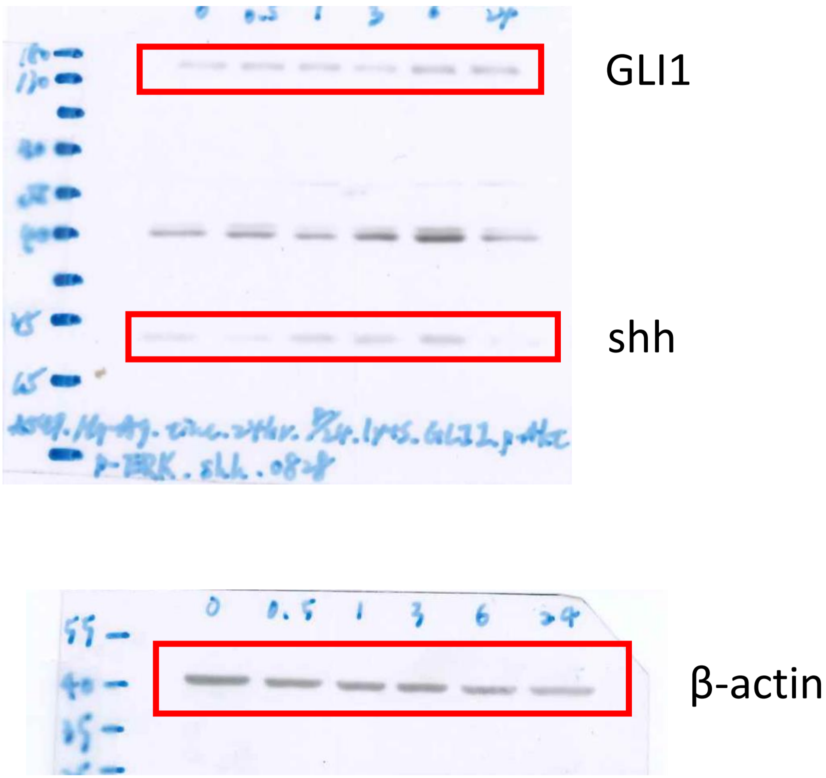

Figure 1C

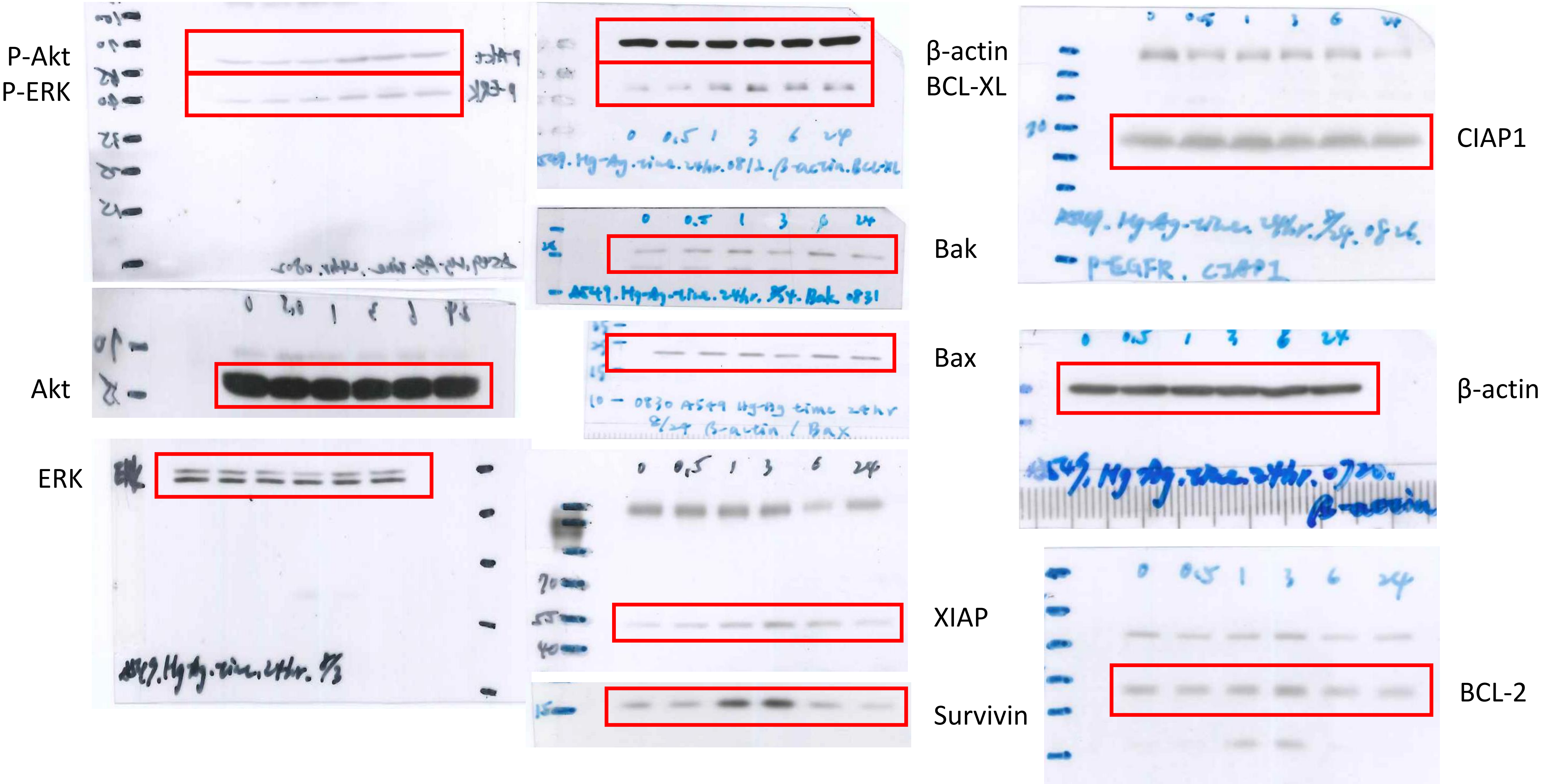
P-Akt
P-ERK
β-actin
BCL-XL
CIAP1
Bak
Akt
Bax
β-actin
ERK
XIAP
Survivin
BCL-2

Figure 2C

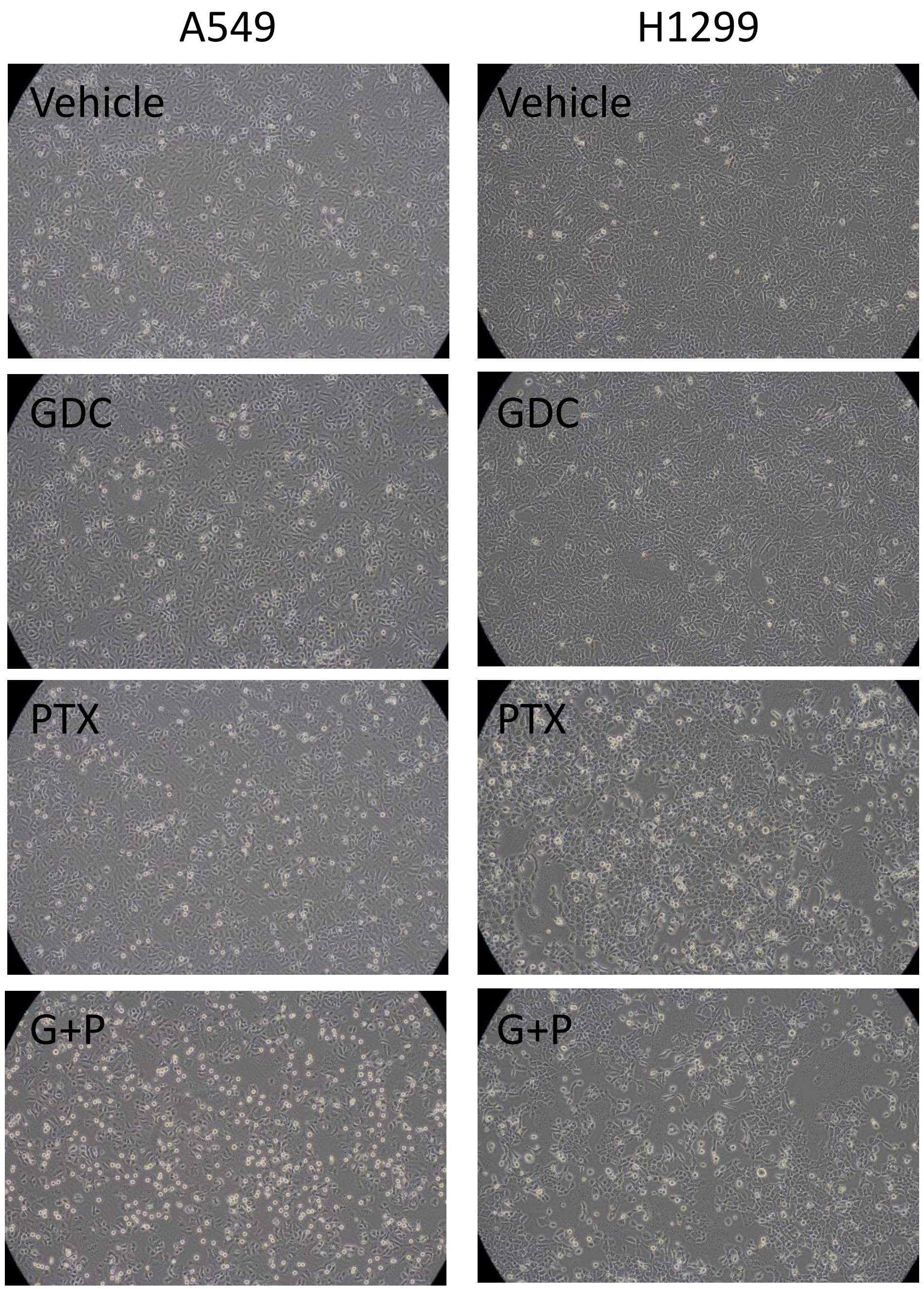

Figure 2F

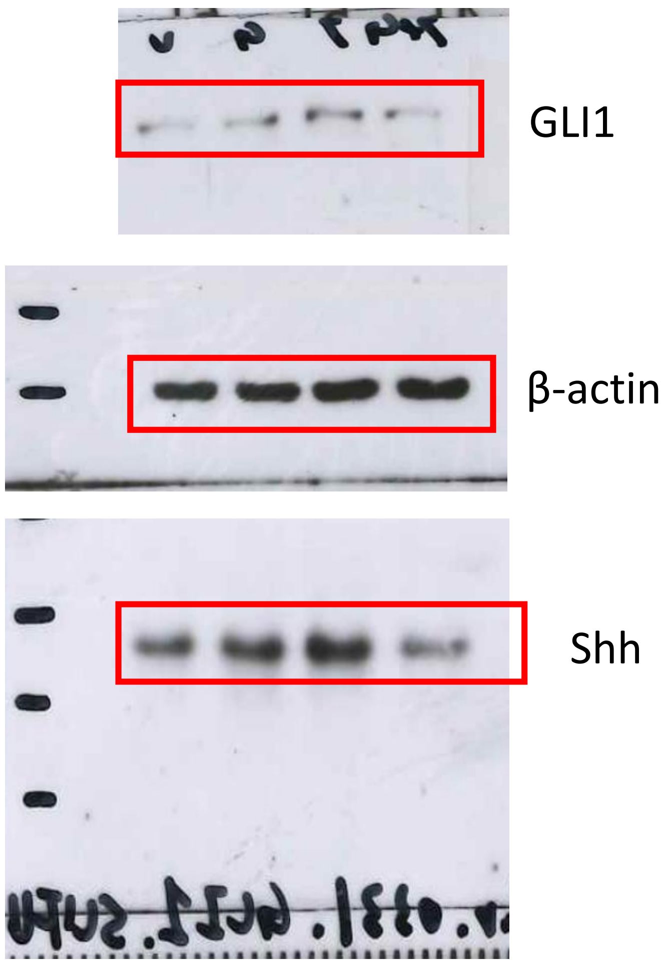

Figure 3A

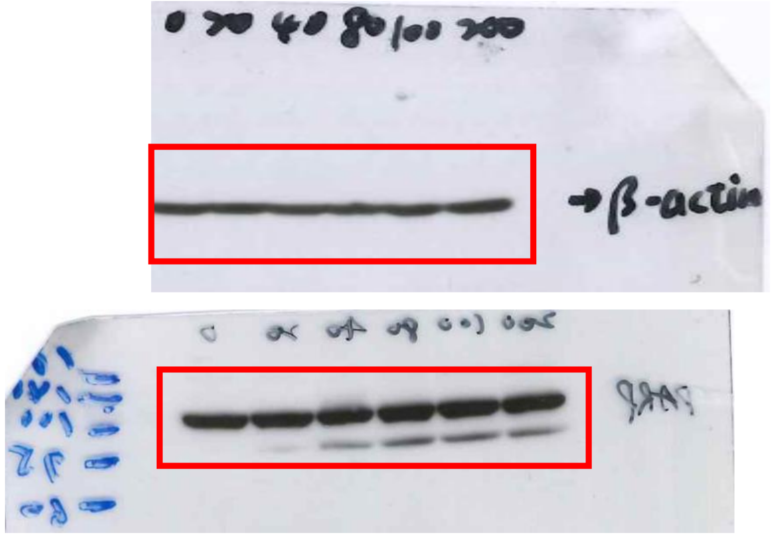

Figure 3B

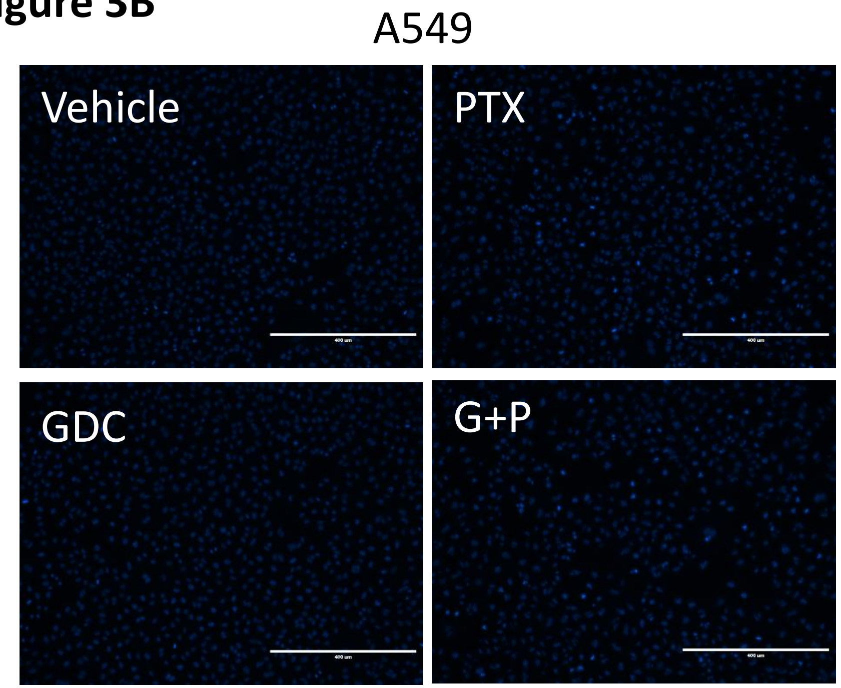

Figure 3C

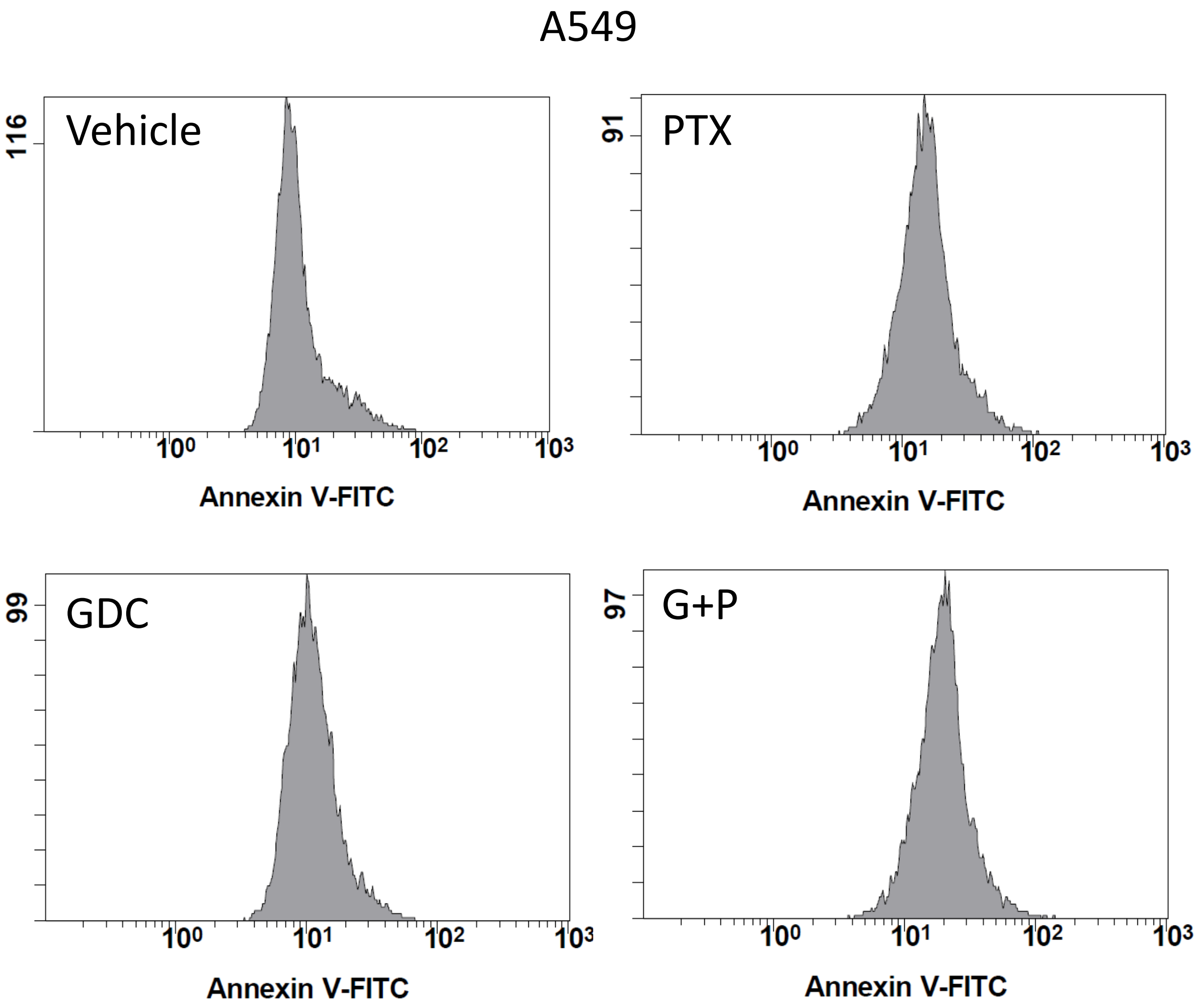

Figure 3D

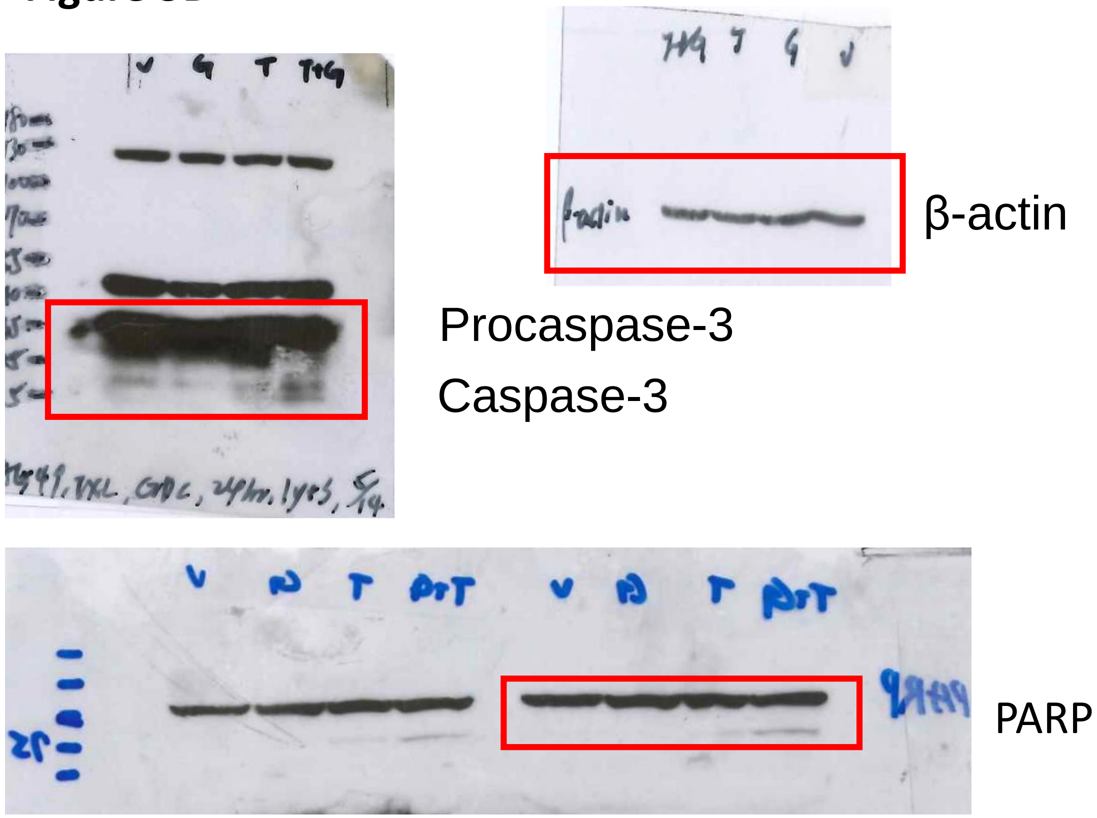

Figure 3F

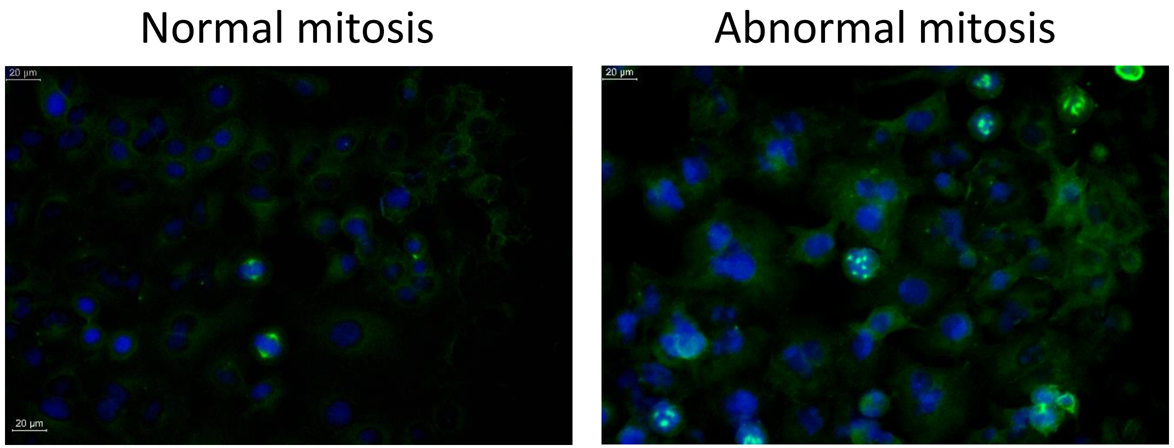

Figure 3G

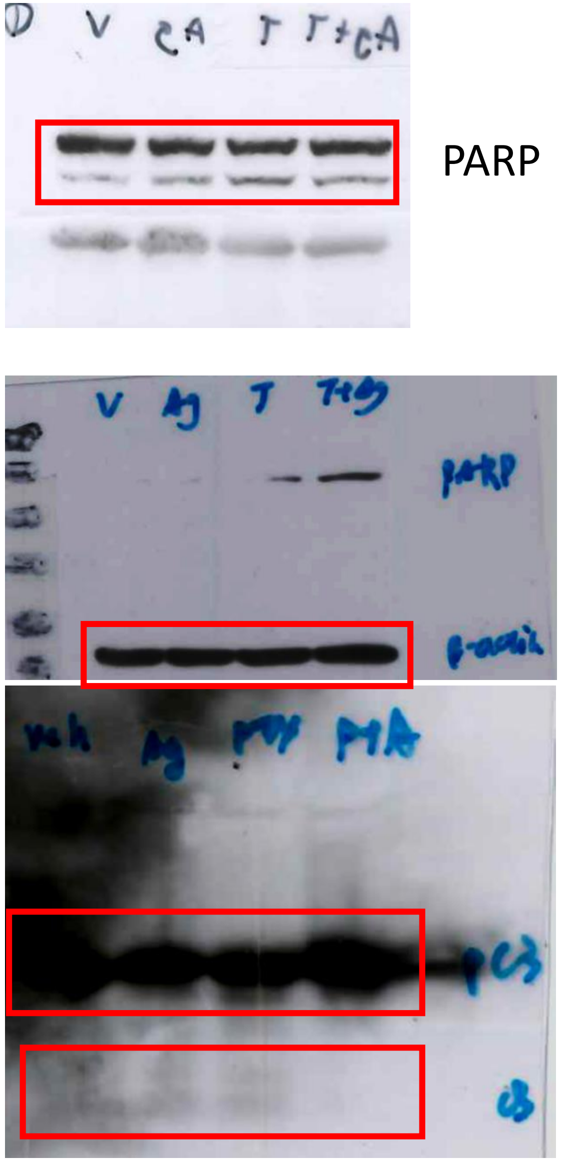

PARP

β-actin

Procaspase-3

caspase-3

Figure 3H

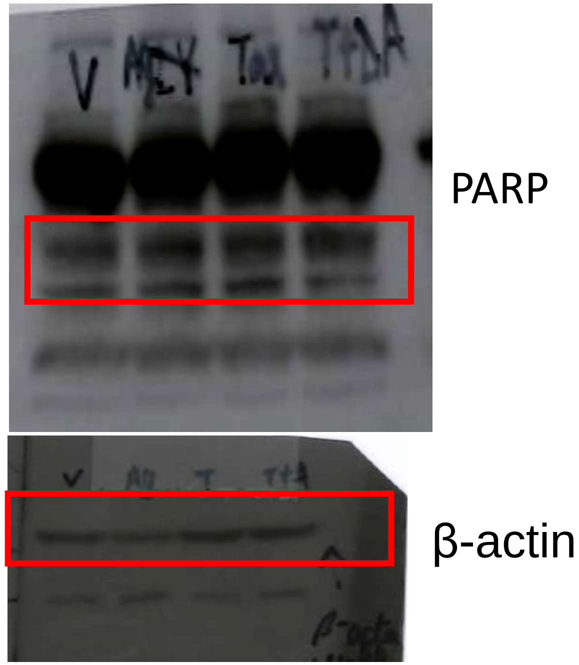

PARP

β-actin

Figure 4A

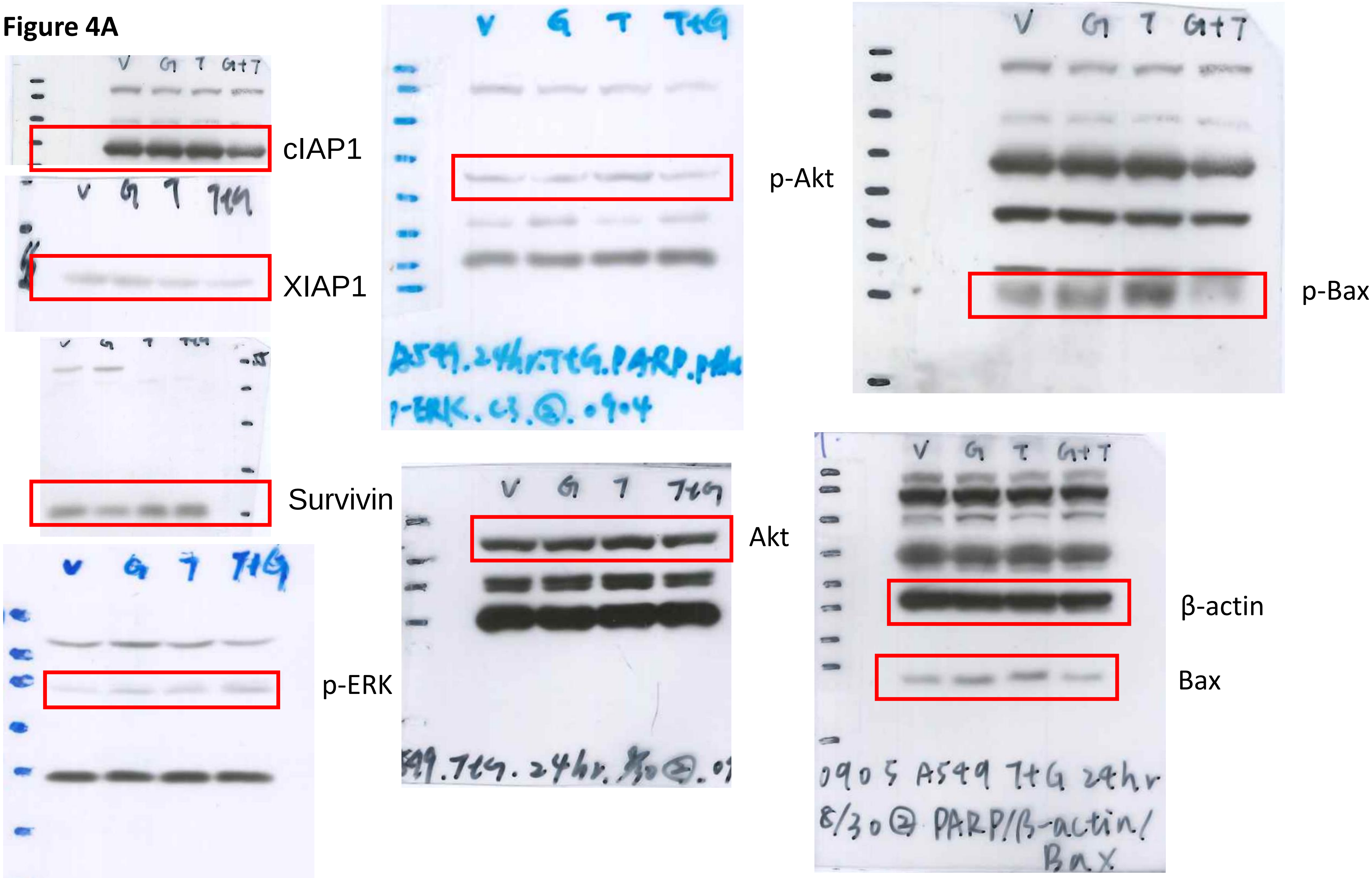

Figure 4B

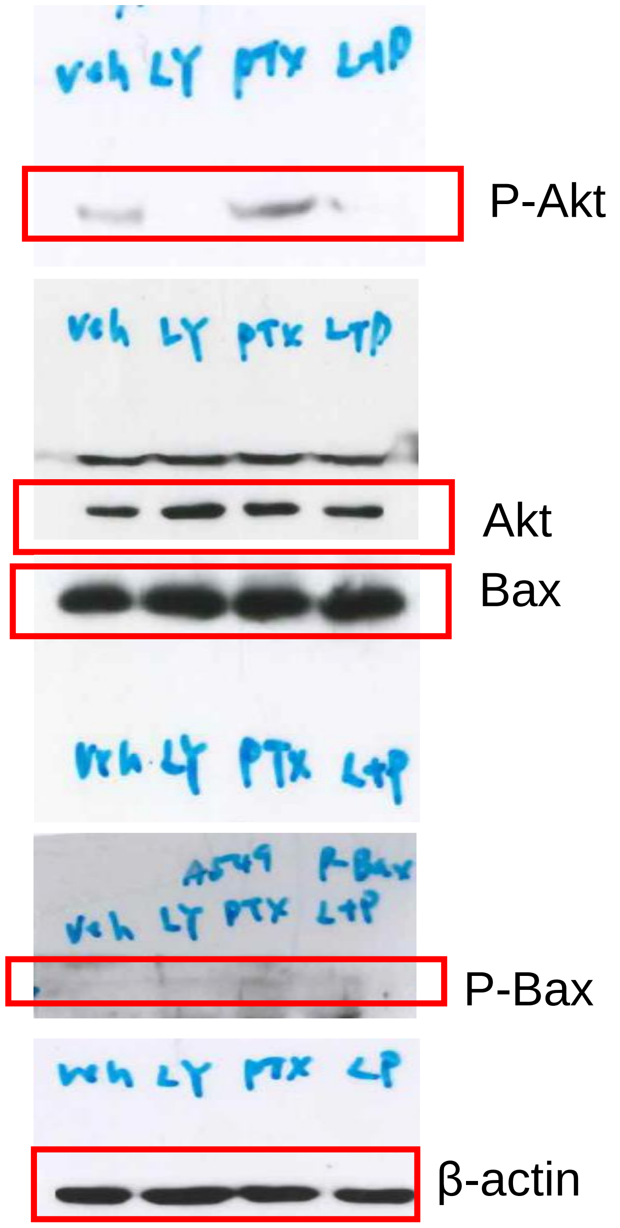

Figure 4C

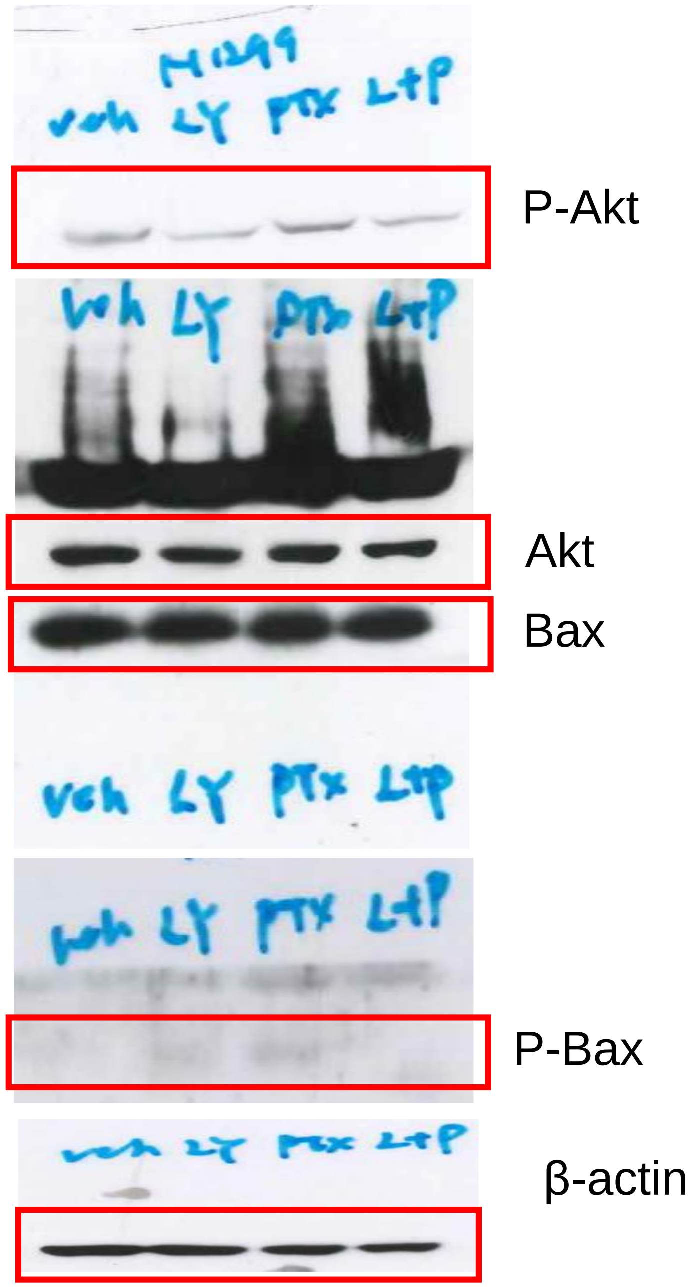

Figure 4D

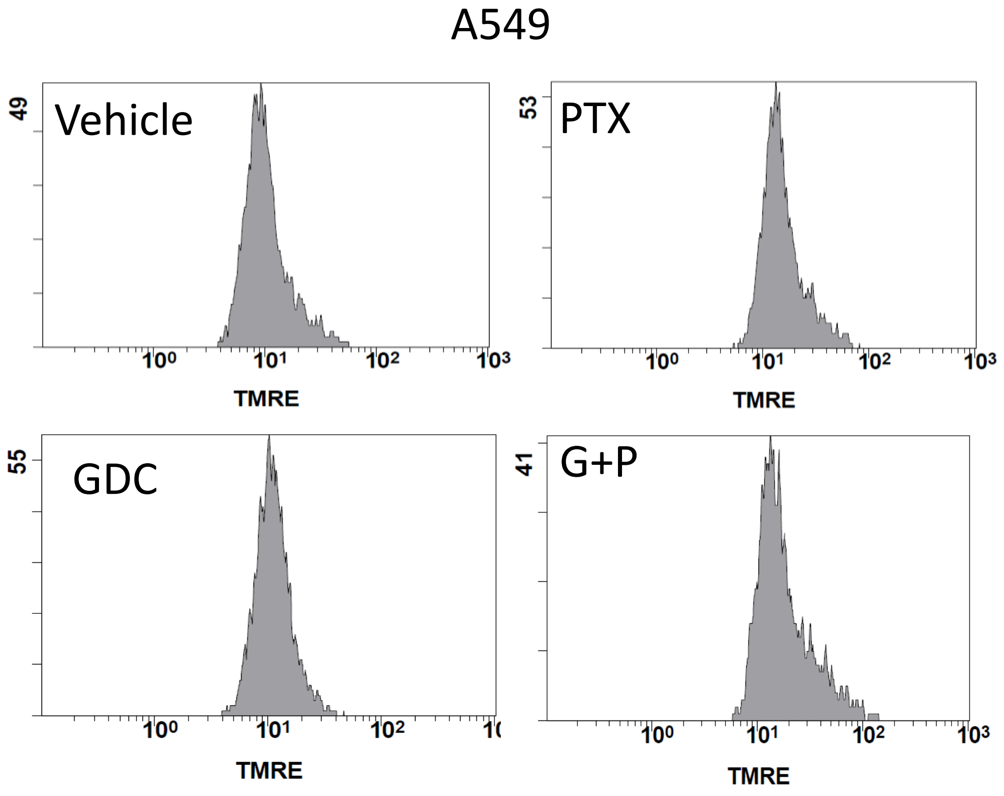

Figure 5A

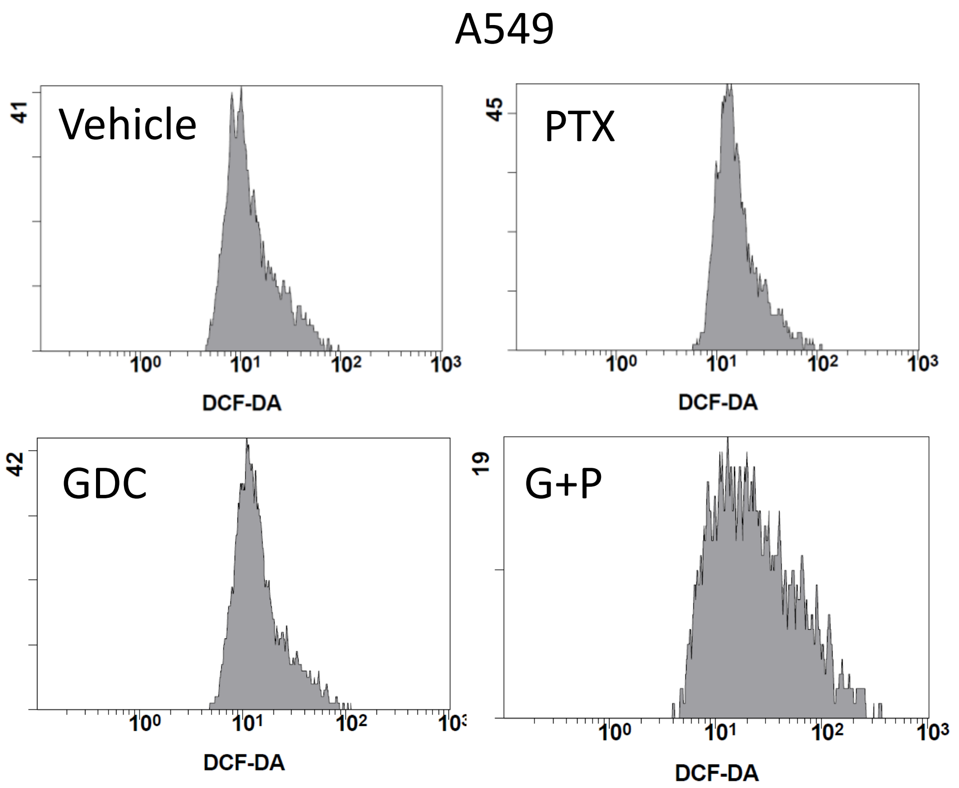

Figure 5B

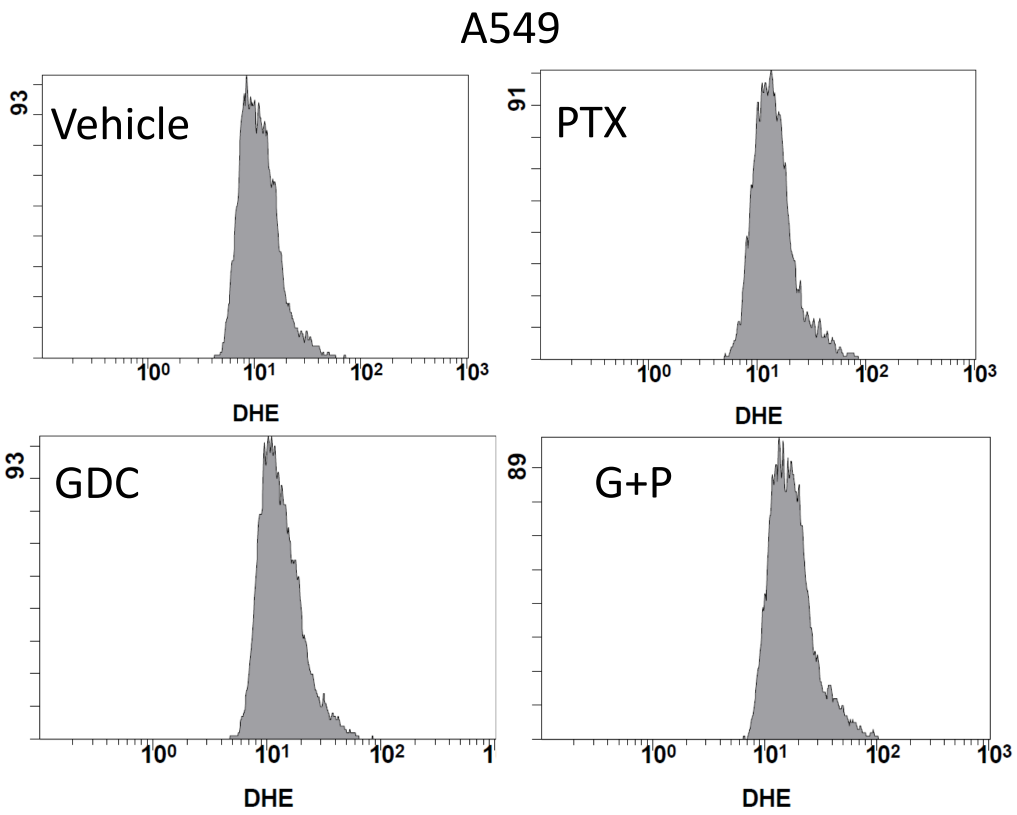

Figure 5C

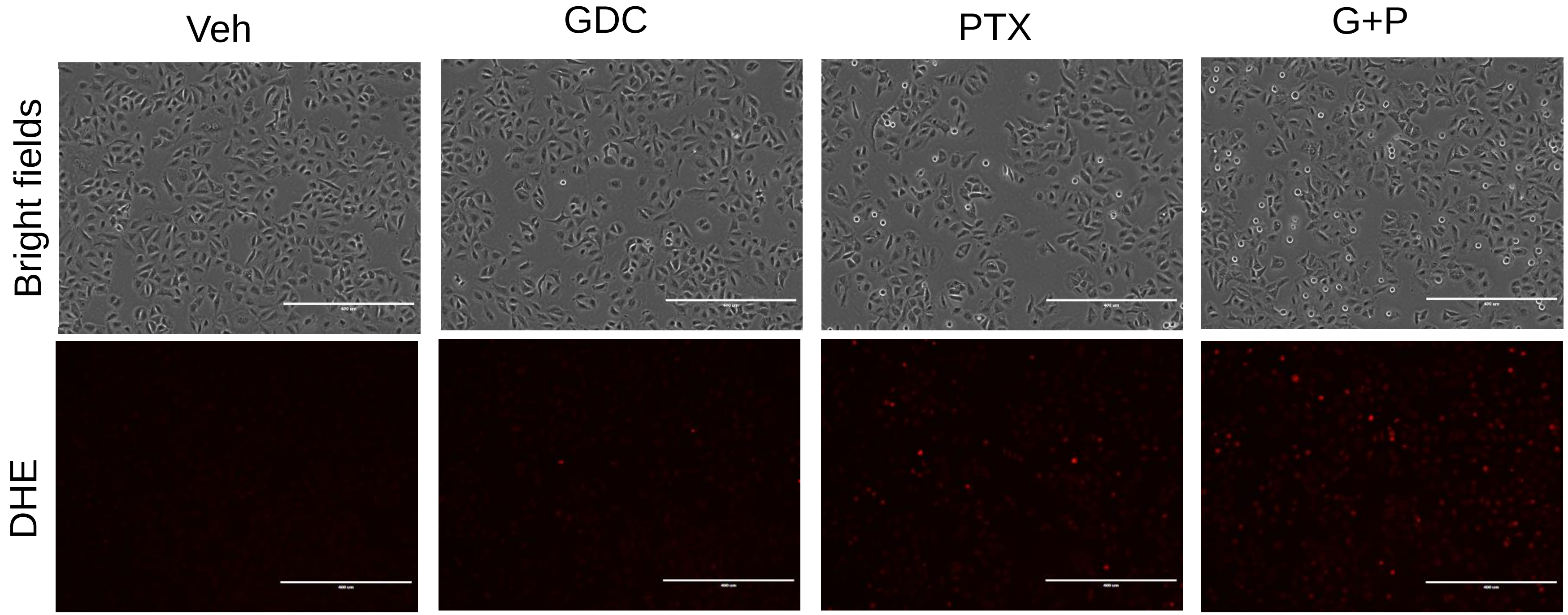

Figure 5F

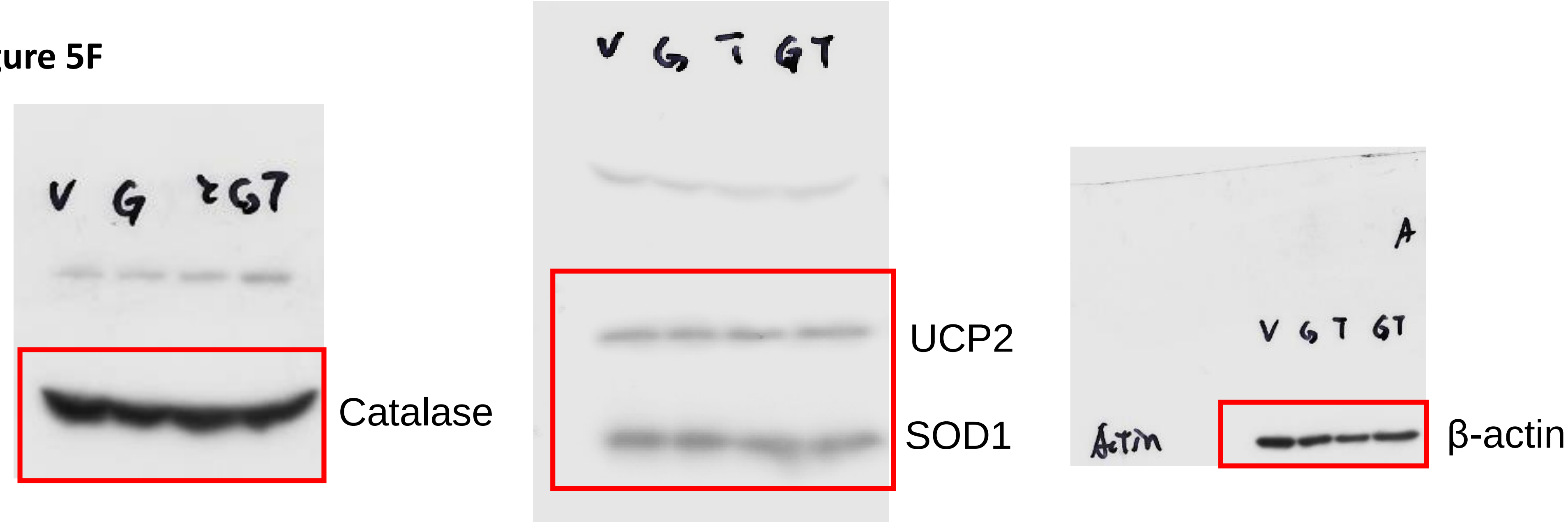

Supplement: Supplementary file 1 [file DataSheet1.PDF]
